# Fitting Copulas with Maximal Entropy

**DOI:** 10.3390/e27010087

**Published:** 2025-01-18

**Authors:** Milan Bubák, Mirko Navara

**Affiliations:** Department of Cybernetics, Faculty of Electrical Engineering, Czech Technical University in Prague, CZ-166 27 Prague, Czech Republic; bubakmil@fel.cvut.cz

**Keywords:** maximum entropy estimator, copula, density, convex optimization

## Abstract

We deal with two-dimensional copulas from the perspective of their differential entropy. We formulate a problem of finding a copula with maximum differential entropy when some copula values are given. As expected, the solution is a copula with a piecewise constant density (a checkerboard copula). This allows us to simplify the optimization of the continuous objective function, the differential entropy, to an optimization of finitely many density values. We present several ideas to simplify this problem. It has a feasible numerical solution. We also present several instances that admit closed-form solutions.

## 1. Formulation of the Task

### 1.1. Motivation

Copulas are a successful tool for the description of *any* dependence of continuous random variables. Based on empirical data, we may try to find a copula that best describes the underlying distribution.

The standard approach to fitting the *parametric copula classes* is widely used across various copula applications. The hidden assumption behind this is the knowledge of the dependence model, for which we try to estimate the parameters. However, there are many applications where this approach is not desirable because of the lack of knowledge, insufficient sample size, or high dimensions of the task. Therefore, we want to find a model based on *general copulas*. When there is no other information than the sample, the empirical or discrete copulas and their continuous extensions can be used. Because of the incomplete information of copula values in between the known values, the maximum entropy principle can also be applied.

We study the problem of finding a joint probability distribution from partial information when we are given the following:

(I1) The marginals;
(I2) The values of the joint cumulative distribution function (*joint cdf*) at finitely many points.

This refers to the situation when we have (or assume to have) complete information about the marginals, but we do not know their dependence. The sample is discrete and can possibly be discretized to a coarser scale. Thus, we know the desired values of the copula only on a *2D rectangular grid* of points resulting from the discretization. This task has a standard solution (a *checkerboard copula*, see below). However, some of these constraints may be missing for various reasons. In particular, we may have too few elements of the sample (possibly none) in some 2D intervals, so we do not consider it a reliable support for modeling the whole copula. We investigate this case herein.

### 1.2. Criteria of Optimality

Suppose that we have two continuous random variables, *X* and *Y*, with known cumulative distribution functions, $F_{X}$ and $F_{Y}$. They need not be independent and their dependence can be specified, e.g., by their joint cdf, $F_{X,Y}$. According to Sklar’s theorem (see [1,2]), this can also be determined by the respective copula, $C_{X,Y}$. This is the joint cumulative distribution function of the transformed random variables $F_{X}\left(X\right)$ and $F_{Y}\left(Y\right)$, which have the uniform distribution on the interval $]0,1[$ (the bounds 0 and 1 are omitted for the simplification of formulas).

$$C_{X,Y}\left(x,y\right)=F_{F_{X}\left(X\right),F_{Y}\left(Y\right)}\left(x,y\right)=F_{X,Y}\left(s,t\right)\;,$$

where *s* and *t* are such that $F_{X}\left(s\right)=x$ and $F_{Y}\left(t\right)=y$. The copula $C_{X,Y}$ gives “pure” information on the dependence of *X* and *Y*, independently of the marginal distributions. It allows for the expression of an arbitrary dependence between two continuous random variables. (More generally, an *n*-copula describes the dependence of *n* random variables. Here, we only deal with 2-copulas.)

**Definition 1.**  *If there is a function $c_{X,Y}$ such that the copula can be expressed as its integral,*

$$C_{X,Y}\left(\bar{x},\bar{y}\right)=\int_{0}^{\bar{x}}\int_{0}^{\bar{y}}c_{X,Y}\left(x,y\right)\;dx\;dy\;,$$

*it is called* adensity *of the copula $C_{X,Y}$. The* differential entropy *of the copula $C_{X,Y}$ is the (Shannon) entropy* [3] *of its density, given by*

(1) $$\mathrm{Ent}\left(c_{X,Y}\right)=\int_{0}^{1}\int_{0}^{1}\eta \left(c_{X,Y}\left(x,y\right)\right)\;dx\;dy\;,$$

*where*

$$\eta \left(w\right):=\left\{\begin{array}{cc} -w\;\ln w & \mathrm{if}\;w>0\;,\\ 0 & \mathrm{if}\;w=0\;. \end{array}\right.$$

**Remark 1.**  *The differential entropy of a copula does not exist if the copula is not absolutely continuous, i.e., it does not have a density. Our proposed solution avoids this problem.*

### 1.3. Related Work and History of the Problem

One of the earliest pioneers in studying multivariate distributions was Claude E. Shannon, who introduced his groundbreaking work on information theory, in which he laid the foundation for the study of dependency structures in multivariate distributions [3].

The principle of maximum entropy in the choice of a distribution was well defended by Jaynes [4]. Several approaches to this aim have been proposed.

The study of copulas in relation to entropy began to gain attention around 2010, with foundational work by Pougaza et al. [5,6]. The authors gave a comprehensive overview and motivation of the maximum entropy principle. The paper [5] deals equally with several definitions of entropy as the best motivated notion, whereas here, we consider only the original Shannon entropy.

The authors maximized the differential entropy of the original *joint cdf*, while we propose to maximize the differential entropy of the corresponding *copula*.

The early contributions were primarily theoretical. Later on, Ma and Sun [7] introduced the use of the maximum entropy principle to estimate mutual information via copulas. Subsequently, Singh and Zhang [8] expanded the application of copulas connected to entropy for multivariate stochastic modeling in water engineering.

Piantadosi et al. [9] (resp., [10]) found bivariate (resp., multivariate) copulas with maximal differential entropy under the knowledge of the following:

(I1) The marginals;
(I3) The grade correlation coefficients.

Their solutions are checkerboard copulas.

Recently, Lin et al. [11] applied the principle of maximum entropy and found checkerboard copulas as solutions. Their approach differs in the aspect that they start from distributions whose marginals are not continuous; the discontinuities determine the checkerboard structure. The difficulties of the extension of the copula approach to distributions with discontinuous marginals are presented by Genest et al. [12].

While the literature on this topic remains relatively limited, this leaves considerable room for further theoretical advancements and potential new applications.

None of the preceding approaches restricts solutions by prescribed values at some points (I2). Sometimes, the values of the joint cdf (and hence, also of the copula) at some points are known. Such constraints can originate, e.g., from a discretization of a continuous scale. We aim to choose a distribution fitting to these constraints.

Let us choose the distribution whose copula has maximal differential entropy (among those fitting to the constraints). The principle of maximal entropy was successfully used in many other tasks. It expresses our intention “not to include more information than that contained in the constraints.” Choosing this option, we try not to introduce any other dependence than that which follows from the given restrictions. This seems to be a natural criterion for the choice of the model fitting to constraints.

The result can be equivalently described by the joint cdf or by the copula. The differential entropy of the joint cdf suffers some drawbacks, e.g., it is not invariant to the change of scale; the entropies of random variables *X* and cX, c≠1, are different. In contrast to it, the copula remains the same even if the random variables are transformed by arbitrary increasing functions f,g,

$$C_{X,Y}\left(x,y\right)=C_{f(X),g(Y)}\left(x,y\right)\;.$$

Thus the differential entropy of a copula is a well-defined notion which can be a criterion for the choice of the model. (We acknowledge that Michal Dibala came up with the idea of using maximum entropy as a criterion for the choice of a copula [13]. The present paper explores this idea in detail; it is based on the bachelor thesis [14]).

In this paper, we formulate the task of finding a copula with maximal differential entropy fitting the constraints. We convert it to a finite-dimensional optimization problem. As one of the main contributions, we prove that the result is independent of some inputs. This further simplifies the task. It leads to a system of higher-order polynomial equations which allows for a numerical solution using convex optimization. We show that in the simplest cases, an analytical solution is also feasible.

### 1.4. Notation and Formulation of the Problem

Before the formulation of the task solved in this paper, let us introduce our notation. We restrict attention to 2-copulas, i.e., *binary* functions describing the dependence of *two* random variables. We aim to find a copula, $C_{X,Y}:{\left[0,1\right]}^{2}\to \left[0,1\right]$. As we shall not deal with another copula in the sequel, we shall denote it briefly by *C*. For the readers not familiar with the notion of a copula, it can be any joint cdf with marginals uniformly distributed on $]0,1[$. (Strictly speaking, the joint cdf is defined on the whole plane, but its restriction to the unit square, even to its interior, ${]0,1[}^{2}$, determines the copula uniquely.)

They are characterized by the following necessary and sufficient conditions:

(2) $$\begin{array}{cc} C(w,0)=C(0,w) & =0\;, \end{array}$$

(3) $$\begin{array}{cc} C(w,1)=C(1,w) & =w\;, \end{array}$$

(4) $$\begin{array}{cc} C\left(x_{i},y_{j}\right)-C\left(x_{i},y_{\ell }\right)-C\left(x_{k},y_{j}\right)+C\left(x_{k},y_{\ell }\right) & \geq 0\;\;\mathrm{for}\;x_{k}<x_{i},\;y_{\ell }<y_{j}\;. \end{array}$$

Inequality (4) means that the probability

(5) $$P\left(x_{k}<X<x_{i},\;y_{\ell }<Y<y_{j}\right)=C\left(x_{i},y_{j}\right)-C\left(x_{i},y_{\ell }\right)-C\left(x_{k},y_{j}\right)+C\left(x_{k},y_{\ell }\right)$$

is non-negative.

The constraints are finitely many points in ${]0,1[}^{2}$ where the value of the copula is given. Their first (resp., second) coordinates can be organized in an increasing sequence $\left(x_{1},\ldots ,x_{I-1}\right)$, resp., $\left(y_{1},\ldots ,y_{J-1}\right)$. These values determine a rectangular grid. At some of the grid points, $\left(x_{i},y_{j}\right)$, the values $z_{i,j}$ are given:

(6) $$C\left(x_{i},y_{j}\right)=z_{i,j}\;.$$

We denote by $G_{0}$ the set of all indices (i,j)∈{1,…,I−1}×{1,…,J−1} for which the values $z_{i,j}$ of the copula are given by (6). To simplify the notation, we additionally define $x_{0}=y_{0}=0$, $x_{I}=y_{J}=1$. The corresponding new grid points are at the boundary of the domain of the copula and form the set

$$B_{I,J}=\left\{\left(x_{i},0\right),\left(x_{i},1\right)|i=0,\ldots ,k\right\}\cup \left\{\left(0,y_{j}\right),\left(1,y_{j}\right)|j=0,\ldots ,J\right\}\;.$$

The values of the copula at the boundary are given by (2) and (3), so we may define $z_{i,j}$ for all $\left(i,j\right)\in B_{I,J}$ by

(7) $$\begin{array}{cccc} z_{i,0} & =C(x_{i},0)=0\;, & z_{0,j} & =C(0,y_{j})=0\;, \end{array}$$

(8) $$\begin{array}{cccc} z_{i,J} & =C\left(x_{i},1\right)=x_{i}\;, & z_{I,j} & =C\left(1,y_{j}\right)=y_{j}\;. \end{array}$$

In total, (6) is required for all (i,j) from the set

$$G=G_{0}\cup B_{I,J}$$

and $G_{0}$ can be expressed as

$$G_{0}=G\setminus B_{I,J}=G\cap {]0,1[}^{2}\;.$$

**Figure 1:**
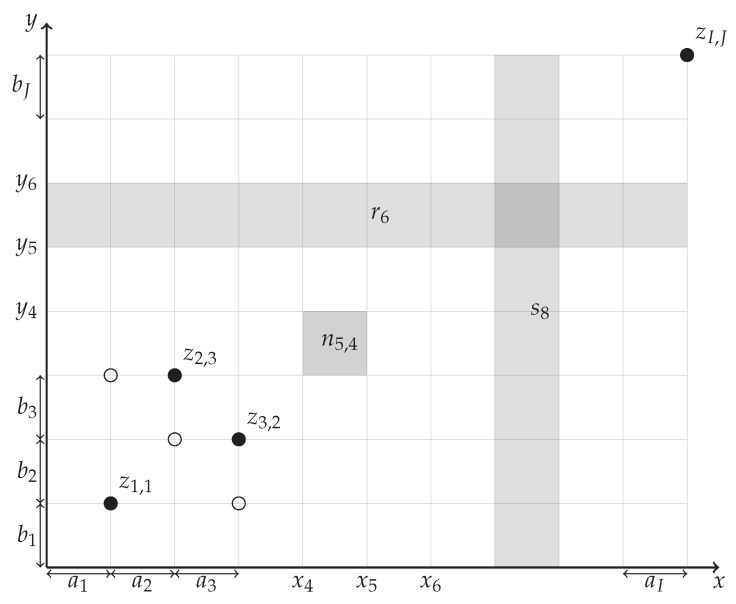
Sample of our notation.

**Figure 2:**
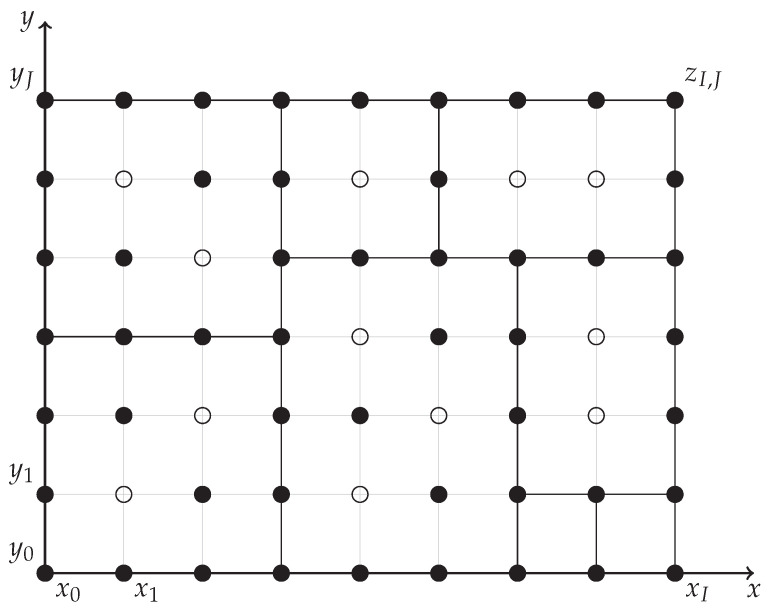
Possible set *G* of points in which the values of a copula are given and a covering of the grid by disjoint irreducible rectangles (bold lines).

In the following figures, we use a filled disk to denote a grid point at which the value is given (an element of *G*) and an empty circle to denote a grid point where the value is not restricted. Figure 1 and Figure 2 demonstrate our notation.

**Problem 1.**  *Let $0=x_{0}<x_{1}<\cdots <x_{I}=1$, $0=y_{0}<y_{1}<\cdots <y_{J}=1$, and let there be given values $z_{i,j}\in \left[0,1\right]$ for all (i,j) in some set G such that $B_{I,J}\subseteq G\subseteq \left\{0,\ldots ,I\right\}\times \left\{0,\ldots ,J\right\}$. Find a copula C satisfying *(6)* for all (i,j)∈G. Moreover, among all such copulas, we want to find one with maximum differential entropy.*

The necessary and sufficient conditions for a copula, in particular (4), imply necessary conditions for the solvability of Problem 1, which will later also appear as sufficient.

**Proposition 1.**  *A necessary condition for the existence of a solution to Problem 1 is the conjunction of *(7), (8)*, and*

(N) *If (i,j),(i,ℓ),(k,j),(k,ℓ)∈G, k<i, ℓ<j, then $z_{i,j}-z_{i,\ell }-z_{k,j}+z_{k,\ell }\geq 0\;.$*

In the sequel, we assume that the constraints satisfy these conditions.

## 2. General Results

### 2.1. Reformulation to a Finite-Dimensional Optimization

As formulated, Problem 1 looks like a task from variational calculus. We shall transform it into a finite-dimensional optimization problem.

It is well known that among all absolutely continuous distributions on a bounded set, the uniform distribution has the highest differential entropy (if the integral of the density over the whole domain is fixed). We shall derive a consequence of this principle. To simplify its formulation, we denote the lengths of intervals between neighboring grid points:

$$\begin{array}{cc} a_{i} & =x_{i}-x_{i-1}\;,\\ b_{j} & =y_{j}-y_{j-1} \end{array}$$

for all i∈{1,…,I}, j∈{1,…,J} (see Figure 1).

**Proposition 2.**  *Let i∈{1,…,I}, j∈{1,…,J}. The restriction of the copula C to the two-dimensional open interval $]x_{i-1},x_{i}[\times ]y_{j-1},y_{j}[$ contributes to the copula differential entropy by*

(9) $$\int_{x_{i-1}}^{x_{i}}\int_{y_{j-1}}^{y_{j}}\eta \left(c_{X,Y}\left(x,y\right)\right)\;dx\;dy\;.$$

*Among all copulas with a given value*

(10) $$\begin{array}{cc} n_{i,j} & :=C\left(x_{i},y_{j}\right)-C\left(x_{i},y_{j-1}\right)-C\left(x_{i-1},y_{j}\right)+C\left(x_{i-1},y_{j-1}\right)\\ \; & =P(x_{i-1}<X<x_{i},\;y_{j-1}<Y<y_{j})\;, \end{array}$$

*the contribution *(9)* is maximal iff its density is constant,*

(11) $$c_{X,Y}\left(x,y\right)=\frac{n_{i,j}}{\left(x_{i}-x_{i-1}\right)\left(y_{j}-y_{j-1}\right)}=\frac{n_{i,j}}{a_{i}\;b_{j}}\;,$$

*almost everywhere on $]x_{i-1},x_{i}[\times ]y_{j-1},y_{j}[$. Without loss of generality, we may choose the copula with density *(11)* for all $\left(x,y\right)\in ]x_{i-1},x_{i}[\times ]y_{j-1},y_{j}[$.*

Proposition 2 determines the solution to Problem 1 (almost everywhere) when all values

$$C\left(x_{i},y_{j}\right)\;,\;C\left(x_{i},y_{j-1}\right)\;,\;C\left(x_{i-1},y_{j}\right)\;,\;C\left(x_{i-1},y_{j-1}\right)$$

are given, i.e., (i,j), (i,j−1), (i−1,j), (i−1,j−1)∈G. Even when this is not the case, there are some optimal values $n_{i,j}$, (i,j)∈{1,…,I}×{1,…,J}, and the copula density on $\left(x_{i-1},x_{i}\right)\times \left(y_{j-1},y_{j}\right)$ can be chosen according to (11) because there are no other restrictions on its values. This observation allows us to restrict attention to copulas with piecewise constant densities, the so-called *checkerboard copulas*. Lin et al. [11] also found checkerboard copulas by maximization of the differential entropy, although they solved a different task.

The need to find the optimal values of finitely many densities on intervals (rectangles) $]x_{i-1},x_{i}[\times ]y_{j-1},y_{j}[$ for (i,j)∈{1,…,I}×{1,…,J} remains. Equivalently, we look for the values $n_{i,j}$ defined by (10). From them, the values of the copula at grid points can be computed as sums

(12) $$C\left(x_{i},y_{j}\right)=\sum_{u=1}^{i}\sum_{v=1}^{j}n_{u,v}\;.$$

In particular, for i=I, resp., j=J, we obtain

$$\begin{array}{cc} \sum_{u=1}^{I}\sum_{v=1}^{j}n_{u,v} & =C\left(x_{I},y_{j}\right)=C\left(1,y_{j}\right)=y_{j}\;,\\ \sum_{u=1}^{i}\sum_{v=1}^{J}n_{u,v} & =C\left(x_{i},y_{J}\right)=C\left(x_{i},1\right)=x_{i}\;. \end{array}$$

These formulas can be equivalently expressed using row and column sums:

$$\begin{array}{cc} \sum_{u=1}^{I}n_{u,j} & =y_{j}-y_{j-1}\;,\\ \sum_{v=1}^{J}n_{i,v} & =x_{i}-x_{i-1}\;. \end{array}$$

**Remark 2.**  *We do not deal with the values of density on the boundaries of these intervals. The boundaries are of measure zero, so they do not influence the results. In fact, the densities at these boundaries are not uniquely defined.*

*Copulas that do not have densities or entropies (see Remark 1) do not seem to be good candidates for approximation and cannot compete with our proposed solution.*

The differential entropy of the checkerboard copula is

$$\begin{array}{cc} \mathrm{Ent}(c_{X,Y}) & =\int_{0}^{1}\int_{0}^{1}\eta \left(c_{X,Y}\left(x,y\right)\right)\;dx\;dy\\ \; & =\sum_{i=1}^{I}\sum_{j=1}^{J}a_{i}\;b_{j}\;\eta \left(\frac{n_{i,j}}{a_{i}\;b_{j}}\right)\\ \; & =-\sum_{i=1}^{I}\sum_{j=1}^{J}n_{i,j}\;\ln\left(\frac{n_{i,j}}{a_{i}\;b_{j}}\right)\;, \end{array}$$

provided that all $n_{i,j}\neq 0$ (otherwise, the corresponding summand of the differential entropy is 0, i.e., minimal). We shall deal with the following reformulation:

**Problem 2.**  *Let $0=x_{0}<x_{1}<\cdots <x_{I}=1$, $0=y_{0}<y_{1}<\cdots <y_{J}=1$, and let there be given values $z_{i,j}\in \left[0,1\right]$ for all (i,j) in some set G such that $B_{I,J}\subseteq G\subseteq \left\{0,\ldots ,I\right\}\times \left\{0,\ldots ,J\right\}$. Suppose that values $z_{i,j}$, (i,j)∈G, satisfy the necessary conditions from Proposition 1. Find values $n_{i,j}\in \left[0,1\right]$, (i,j)∈{1,…,I}×{1,…,J}, satisfying*

(13) $$\begin{array}{cc} \sum_{u=1}^{I}n_{u,j} & =y_{j}-y_{j-1}\;, \end{array}$$

(14) $$\begin{array}{cc} \sum_{v=1}^{J}n_{i,v} & =x_{i}-x_{i-1}\;, \end{array}$$

(15) $$\begin{array}{cc} \sum_{u=1}^{i}\sum_{v=1}^{j}n_{u,v} & =z_{i,j}\;\mathrm{for}\;\mathrm{all}\;\left(i,j\right)\in G. \end{array}$$

*If i=0 (resp., j=0), then n(0,j) (resp., n(i,0)) are not defined, but the sum from *1* to *0* is considered empty and equal to $0=z_{0,j}$ (resp., $0=z_{i,0}$). Moreover, these values should be chosen such that the differential entropy*

(16) $$\begin{array}{cc} \mathrm{Ent}(c_{X,Y}) & =-\sum_{i=1}^{I}\sum_{j=1}^{J}n_{i,j}\;\ln\left(\frac{n_{i,j}}{\left(x_{i}-x_{i-1}\right)\left(y_{j}-y_{j-1}\right)}\right) \end{array}$$

*is maximal.*

**Remark 3.**  *If G={0,…,I}×{0,…,J}, i.e., if all values $z_{i,j}$, (i,j)∈{1,…,I}×{1,…,J}, are given, then *(10)* and *(6)* determine all $n_{i,j}$ and the solution.*

### 2.2. Decomposition of the Problem

We concentrate on rectangles with the following property:

(B) The values of the copula at all grid points at the boundary of the rectangle $\left[x_{k},x_{K}\right]\times \left[y_{\ell },y_{L}\right]$ are given, i.e.,
  $$\left\{\left(x_{i},y_{\ell }\right),\left(x_{i},y_{L}\right)|i=k,\ldots ,K\right\}\cup \left\{\left(x_{k},y_{j}\right),\left(x_{K},y_{j}\right)|j=\ell ,\ldots ,L\right\}\subseteq G\;.$$

Then, the optimization of the copula inside such a rectangle is independent of the rest of the domain and can be solved separately. For example, in Figure 2, rectangles satisfying (B) are drawn by thick lines. The whole domain [0,1]×[0,1] has the values on the entire boundary given, so it satisfies (B).

Let us consider a rectangle satisfying (B). If all its grid points in some row or column (not at the boundary) are in *G*, then we can split it into two disjoint rectangles satisfying (B) (the choice need not be unique). If this is not the case (i.e., there is a missing value in each row and each column (the boundary rows and columns are not considered)), we call the rectangle *irreducible*. Irreducible rectangles can be equivalently characterized as minimal rectangles satisfying (B). The whole domain can be covered by disjoint irreducible rectangles. This may allow us to decompose the problem into simpler ones, dealing with each irreducible rectangle separately. Figure 2 shows eight irreducible rectangles. Each component of the decomposed task has the following formulation. The active restrictions refer to grid points with indices from the set G∩{k,…,K}×{ℓ,…,L}. Notice that the monotonicity of the values $z_{i,j}$ needs to be formulated explicitly, while in Problem 2, it follows from (7).

**Problem 3.**  *Let $0\leq x_{k}<\cdots <x_{K}\leq 1$, $0\leq y_{\ell }<\cdots <y_{L}\leq 1$, and let G⊆{k,…,K}×{ℓ,…,L} be a set such that*

$$\begin{array}{c} \left\{\left(x_{i},\ell \right),\left(x_{i},L\right)|i=0,\ldots ,I\right\}\cup \left\{\left(k,y_{j}\right),\left(K,y_{j}\right)|j=0,\ldots ,J\right\}\subseteq G\;. \end{array}$$

*Suppose that values $z_{i,j}\in \left[0,1\right]$ are given for all (i,j)∈G so that sequences ${\left(z_{i,\ell }\right)}_{i=k,\ldots ,K}$, ${\left(z_{k,j}\right)}_{j=\ell ,\ldots ,L}$ are nondecreasing and* (N) *is satisfied. Find values $n_{i,j}\in \left[0,1\right]$, (i,j)∈{k+1,…,K}×{ℓ+1,…,L}, satisfying*

(17) $$\begin{array}{cc} \sum_{u=k+1}^{i}\sum_{v=\ell +1}^{j}n_{u,v} & =z_{i,j}-z_{i,\ell }-z_{k,j}+z_{k,\ell } \end{array}$$

*for all (i,j)∈G and such that the contribution to the differential entropy*

(18) $$\begin{array}{c} -\sum_{i=k+1}^{K}\sum_{j=\ell +1}^{L}n_{i,j}\;\ln\left(\frac{n_{i,j}}{\left(x_{i}-x_{i-1}\right)\left(y_{j}-y_{j-1}\right)}\right) \end{array}$$

*is maximal.*

In the sequel, we analyze special cases of irreducible rectangles. Before that, we simplify indexing by shifting the rectangle to the origin.

### 2.3. Shifted Indexing

If a rectangle $\left[x_{k},x_{K}\right]\times \left[y_{\ell },y_{L}\right]$ satisfies (B), the corresponding values $n_{i,j}$, (i,j)∈{k+1,…,K}×{ℓ+1,…,L}, are bounded only by the row and column sums

(19) $$\begin{array}{cc} r_{j} & =\sum_{i=k+1}^{K}n_{i,j}=z_{K,j}-z_{K,j-1}-z_{k,j}+z_{k,j-1}\;,\;j\in \left\{\ell +1,\ldots ,L\right\}\;, \end{array}$$

(20) $$\begin{array}{cc} s_{i} & =\sum_{j=\ell +1}^{L}n_{i,j}=z_{i,L}-z_{i-1,L}-z_{i,\ell }+z_{i-1,\ell }\;,\;i\in \left\{k+1,\ldots ,K\right\}\;, \end{array}$$

which are non-negative values satisfying the equality

(21) $$\sum_{j=\ell }^{L}r_{j}=\sum_{i=k}^{K}s_{i}=\sum_{j=\ell +1}^{L}\sum_{i=k+1}^{K}n_{i,j}=z_{K,L}-z_{k,L}-z_{K,\ell }+z_{k,\ell }\;.$$

The same task is obtained if we take a rectangle of the same size with the lower left vertex at the origin and impose the same requirements on the row and column sums. We shall apply this simplification, and instead of the original task for a general rectangle, we shall, without loss of generality, solve the case when k=ℓ=0 in the above notation (with the new upper bounds still denoted by K,L; the above general indexing will not be used anymore). The boundary grid points form the set

$$B_{K,L}=\left\{\left(x_{i},0\right),\left(x_{i},L\right)|i=0,\ldots ,I\right\}\cup \left\{\left(0,y_{j}\right),\left(K,y_{j}\right)|j=0,\ldots ,J\right\}\;.$$

The situation simplifies not only in indexing (starting from 0, resp., 1), but also the values at the bottom and left edges, which become zero. The values at the top and right bound must be modified to general values $z_{i,L}$, $z_{K,j}$, which are not determined by $x_{i}$ and $y_{j}$ like they were in (8). The modified task is as follows.

**Problem 4.**  *Let $0=x_{0}<\cdots <x_{K}\leq 1$, $0=y_{0}<\cdots <y_{L}\leq 1$, and let there be given values $z_{i,j}\in \left[0,1\right]$ for all (i,j) in some set G such that $B_{K,L}\subseteq G\subseteq \left\{0,\ldots ,K\right\}\times \left\{0,\ldots ,L\right\}$. (From now on, we denote this set again by G, although this was used for all given values from {0,…,I}×{0,…,J} before.) Suppose that values $z_{i,j}$, (i,j)∈G, satisfy the necessary conditions *(N)* and (7) for i∈{0,…,K} and j∈{0,…,L}. Find values $n_{i,j}\in \left[0,1\right]$, (i,j)∈{1,…,K}×{1,…,L}, satisfying*

(22) $$\begin{array}{cc} \sum_{u=1}^{i}\sum_{v=1}^{j}n_{u,v} & =z_{i,j}\;\mathrm{for}\;\mathrm{all}\;\left(i,j\right)\in G\cap ]0,K]\times ]0,L] \end{array}$$

*and such that the contribution to the differential entropy*

(23) $$\begin{array}{c} -\sum_{i=1}^{K}\sum_{j=1}^{L}n_{i,j}\;\ln\left(\frac{n_{i,j}}{\left(x_{i}-x_{i-1}\right)\left(y_{j}-y_{j-1}\right)}\right) \end{array}$$

*is maximal.*

As in [11], this is an optimization of a concave function that has a maximum that can be found numerically (applying standard convex optimization with the opposite sign). For numerical solutions of convex tasks, standard references are, e.g., [15,16,17,18]. We shall show that analytical solutions exist in some cases and we demonstrate them to support the intuition about the task.

## 3. Less General Tasks

In this section, we collect results related to Problem 4. Throughout this section, we consider an irreducible rectangle $\left[0,x_{K}\right]\times \left[0,y_{L}\right]$.

### 3.1. Rectangle with No Given Values Inside

Suppose that the set $G=B_{K,L}$ has an empty intersection with the interior of the rectangle $]0,x_{K}[\times ]0,y_{L}[$. This is an instance of Problem 4; Equation (22) reduces to monotonicity.

**Problem 5.**  *Let $0=x_{0}<\cdots <x_{K}\leq 1$, $0=y_{0}<\cdots <y_{L}\leq 1$, and let*

$$G=\left\{\left(x_{i},0\right),\left(x_{i},L\right)|i=0,\ldots ,I\right\}\cup \left\{\left(0,y_{j}\right),\left(K,y_{j}\right)|j=0,\ldots ,J\right\}\;.$$

*Suppose that values $z_{i,j}\in \left[0,1\right]$ are given for all (i,j)∈G so that *(7)* is satisfied for all i∈{0,…,K} and j∈{0,…,L} and sequences ${\left(z_{i,L}\right)}_{i=0}^{K}$, ${\left(z_{K,j}\right)}_{j=0}^{L}$ are nondecreasing. Find values $n_{i,j}\in \left[0,1\right]$, (i,j)∈{1,…,K}×{1,…,L}, satisfying*

(24) $$\begin{array}{cc} \sum_{u=1}^{K}n_{u,j} & =z_{K,j}\;, \end{array}$$

(25) $$\begin{array}{cc} \sum_{v=1}^{L}n_{i,v} & =z_{i,L}\;, \end{array}$$

*and such that the contribution to the differential entropy*

(26) $$\begin{array}{c} -\sum_{i=1}^{K}\sum_{j=1}^{L}n_{i,j}\;\ln\left(\frac{n_{i,j}}{\left(x_{i}-x_{i-1}\right)\left(y_{j}-y_{j-1}\right)}\right) \end{array}$$

*is maximal.*

The solution need not be a copula with a constant density at $]0,x_{K}[\times ]0,y_{L}[$ because we must keep the correct row and column sums.

We use Lagrange multipliers $\lambda_{1},\ldots ,\lambda_{K}$, $\mu_{1},\ldots ,\mu_{L}$; the Lagrange function is

$$L\left(n_{i,j},\lambda_{i},\mu_{j}\right)=\sum_{i=1}^{K}\sum_{j=1}^{L}-n_{i,j}\ln\frac{n_{i,j}}{a_{i}b_{j}}+\sum_{i=1}^{K}\lambda_{i}\left(\sum_{j=1}^{L}n_{i,j}-s_{i}\right)+\sum_{j=1}^{L}\mu_{j}\left(\sum_{i=1}^{K}n_{i,j}-r_{j}\right)\;.$$

We put all partial derivatives equal to zero and obtain a system of equations

$$\frac{\partial L}{\partial n_{i,j}}=-1-\ln\frac{n_{i,j}}{a_{i}b_{j}}+\lambda_{i}+\mu_{j}=0\;,$$

where i∈{1,2⋯,K}, j∈{1,2⋯,L}. Equivalently,

$$\ln\frac{n_{i,j}}{a_{i}b_{j}}=-1+\lambda_{i}+\mu_{j}\;.$$

Subtracting the last ones, we obtain

$$\begin{array}{cc} \ln\frac{n_{i,j}}{a_{i}b_{j}}-\ln\frac{n_{K,j}}{a_{K}b_{j}} & =\lambda_{i}-\lambda_{K}\;,\\ \ln\frac{n_{i,j}}{a_{i}b_{j}}-\ln\frac{n_{i,L}}{a_{i}b_{L}} & =\mu_{i}-\mu_{L}\;, \end{array}$$

and express $n_{i,j}$ using $n_{K,j}$ or $n_{i,L}$ as

$$\begin{array}{cc} n_{i,j} & =n_{K,j}\;\frac{a_{i}}{a_{K}}\;\exp\left(\lambda_{i}-\lambda_{K}\right)\;,\\ n_{i,j} & =n_{i,L}\;\frac{b_{j}}{b_{L}}\;\exp\left(\mu_{j}-\mu_{L}\right)\;. \end{array}$$

We see that all rows (resp., columns) are multiples of the last one and the matrix composed of all $n_{i,j}$ is a dyad (a product of two vectors),

(27) $$\left[\begin{array}{ccc} n_{1,1} & \cdots & n_{1,K}\\ \vdots & \ddots & \vdots \\ n_{L,1} & \cdots & n_{L,K} \end{array}\right]=c\left[\begin{array}{c} r_{1}\\ \vdots \\ r_{m} \end{array}\right]\left[\begin{array}{ccc} s_{1} & \cdots & s_{t} \end{array}\right]\;,$$

where $r_{j}$ are the row sums, $s_{i}$ the column sums, and the constant *c* can be determined from the known total sum

$$\begin{array}{cc} c\sum_{j=1}^{L}\sum_{i=1}^{K}s_{i}r_{j} & =\sum_{j=1}^{L}r_{j}=\sum_{i=1}^{K}s_{i}\;,\\ c & =\frac{\sum_{j=1}^{L}r_{j}}{\sum_{i=1}^{L}\sum_{j=1}^{K}s_{j}r_{i}}=\frac{1}{\sum_{i=1}^{K}s_{i}}\;. \end{array}$$

This is an expected result that says that the probability density of a result in an elementary rectangle is the product of the probability densities of the marginal distributions.

### 3.2. Methodology of Solving Particular Cases

Here, we collect ideas used to solve particular cases described in the appendices. We consider Problem 4. This leads to a system of linear equations; after the Gauss–Jordan elimination, it could look like

$$M=\left[\begin{array}{ccccccccc} 1 & 0 & 1 & 0 & 0 & \cdots & 0 & 1 & C_{1}\\ 0 & 1 & 1 & 0 & 0 & \cdots & 0 & 0 & C_{2}\\ 0 & 0 & 0 & 1 & 0 & \cdots & 0 & 1 & C_{3}\\ 0 & 0 & 0 & 0 & 1 & \cdots & 0 & 1 & C_{4}\\ \vdots & \vdots & \vdots & \vdots & \vdots & \ddots & \vdots & \vdots & \vdots \\ 0 & 0 & 0 & 0 & 0 & \cdots & 1 & 1 & C_{N} \end{array}\right]\;,$$

where $C_{1},\ldots ,C_{N}$ are some constants computed from the values $z_{i,j}$. Until we consider the maximized differential entropy, some variables $n_{i,j}$ can be chosen and the rest can be computed from them. This describes all possible solutions to the system of linear equations (ignoring their bounds). To maximize the differential entropy, we compute the partial derivatives of the contribution to the differential entropy and set them equal to zero. We obtain

N=KL−cardG=KL−K−L+1−o

equations, where $o=\mathrm{card}(G\cap ]0,x_{K}[\times ]0,y_{L}[)$ is the number of given values inside the rectangle.

**Theorem 1.**  *The optimal solution to Problem 4 is independent of the values $a_{1},\ldots ,a_{K}$, $b_{1},\ldots ,b_{L}$.*

**Proof.**  Recall that

(28) $$n_{i,j}=z_{i,j}-z_{i-1,j}-z_{i,j-1}+z_{i-1,j-1}\;.$$

This leads to an optimization in those variables $z_{i,j}=C(x_{i},y_{j})$ where the copula values are not given ((i,j)∉G).

In the criterion, each variable $z_{i,j}$, $\left(i,j\right)\in G\setminus B_{K,L}$, occurs in four equations: for $n_{i,j}$ and $n_{i+1,j+1}$ with coeefficient 1 and for $n_{i+1,j}$ and $n_{i,j+1}$ with coeefficient −1.

We solve this task by computing the partial derivatives with respect to unknown values $z_{i,j}$ and making them zero:

(29) $$\begin{array}{c} \ln\frac{z_{i,j}-z_{i-1,j}-z_{i,j-1}+z_{i-1,j-1}}{a_{i}b_{j}}+1-\ln\frac{z_{i,j+1}-z_{i-1,j+1}-z_{i,j}+z_{i-1,j}}{a_{i}b_{j+1}}-1\\ -\ln\frac{z_{i+1,j}-z_{i,j}-z_{i+1,j-1}+z_{i,j-1}}{a_{i+1}b_{j}}-1+\ln\frac{z_{i+1,j+1}-z_{i+1,j}-z_{i,j+1}+z_{i,j}}{a_{i+1}b_{j+1}}+1=0\;. \end{array}$$

This can be expressed as

$$\begin{array}{cc} \; & \frac{z_{i,j}-z_{i-1,j}-z_{i,j-1}+z_{i-1,j-1}}{a_{i}b_{j}}\cdot \frac{z_{i+1,j+1}-z_{i+1,j}-z_{i,j+1}+z_{i,j}}{a_{i+1}b_{j+1}}\\ = & \frac{z_{i,j+1}-z_{i-1,j+1}-z_{i,j}+z_{i-1,j}}{a_{i}b_{j+1}}\cdot \frac{z_{i+1,j}-z_{i,j}-z_{i+1,j-1}+z_{i,j-1}}{a_{i+1}b_{j}}\;. \end{array}$$

Multiplying this by $a_{i}b_{j}a_{i+1}b_{j+1}$, we obtain an equation independent of $a_{i}$, $b_{j}$, $a_{i+1}$, and $b_{j+1}$. This holds for all $z_{i,j}$ and $n_{i,j}$, (i,j)∈{1,…,K−1}×{1,…,L−1}∖G. Due to (28), also the values $n_{i,j}$ are independent of $a_{1},\ldots ,a_{K}$, $b_{1},\ldots ,b_{L}$. □

Besides the independence of the result on interval lengths $a_{1},\ldots ,a_{K}$, $b_{1},\ldots ,b_{L}$, we proved that each equation in the system describing entropy maximization is of the form

$$\prod_{\kappa_{1}\in Z_{+}}\left(n_{i,j}+B_{\kappa_{1}}\right)=\prod_{\kappa_{2}\in Z_{-}}\left(-n_{i,j}+B_{\kappa_{2}}\right)\;,$$

where $Z_{+}$ is the set of indices (i,j) for which $n_{i,j}$ occurs with the positive sign, and $Z_{-}$ is the set of indices for which $n_{i,j}$ occurs with the negative sign. Apparently, $\mathrm{card}Z_{+}=\mathrm{card}Z_{-}$; therefore, the units in (29) are canceled.

The only problem is that $n_{i,j}$ may also depend on other variables. Thus, the summands $B_{*}$ contain not only constants $C_{*}$, but also other independent variables.

In Appendix A and Appendix B, we present explicit solutions to some specific instances of Problem 4. They lead to higher-order algebraic equations.

## 4. Conclusions

We formulated the task of fitting a continuous copula to finitely many given values in such a way that the entropy of the copula density is maximal. This is motivated by situations when some of the values are prescribed and the rest of the copula should be “as independent as possible”, with the intention not to include any other dependence than that contained in the constraints. We simplified the task with several hints, showed that it has a unique solution (because it is equivalent to a convex optimization problem), and demonstrated that a closed-form solution can be found analytically in some simple cases, although it may require solving higher-order polynomials. We propose this concept as an alternative to current approaches, which also maximize the entropy but use different restrictions of the admitted copulas.

## Figures and Tables

**Figure A1:**
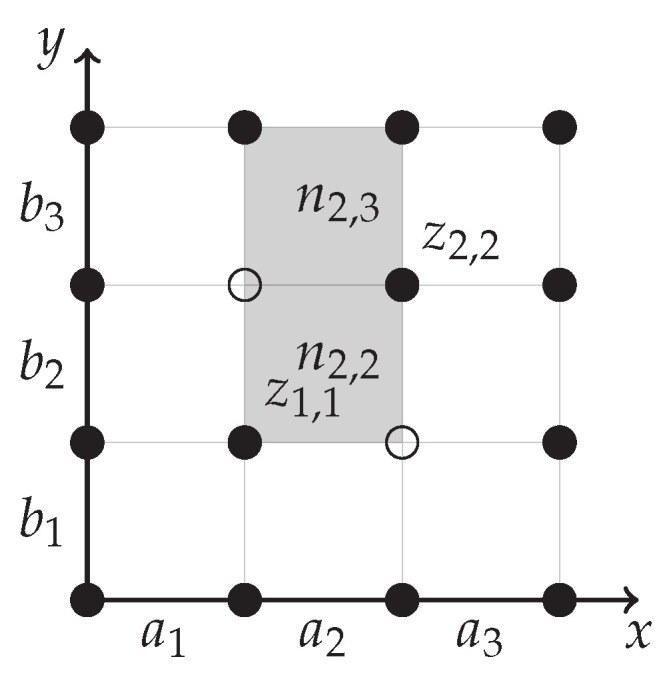
Example 1.

**Figure A2:**
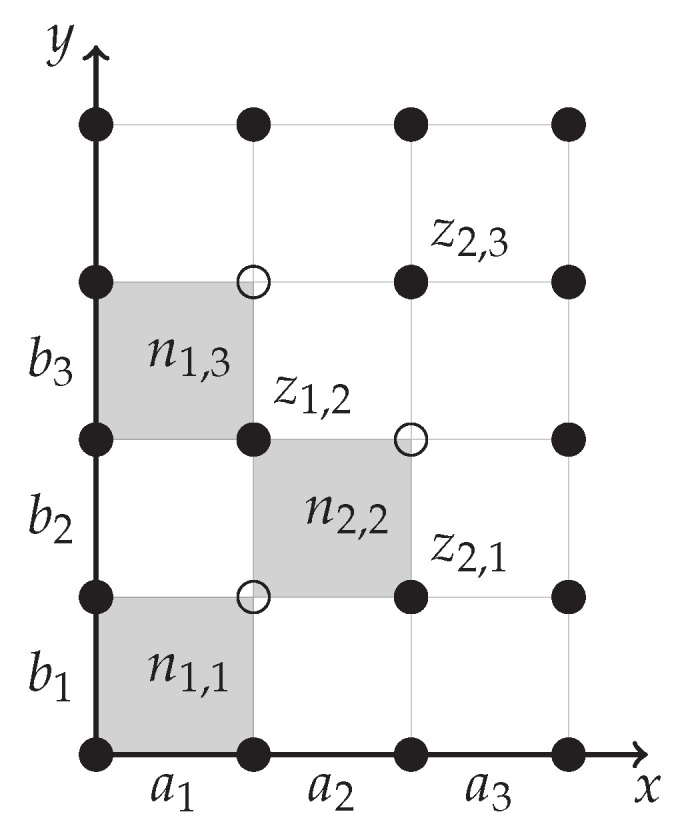
Example 3.

## Data Availability

This research is not accompanied by any additional data.

## Appendix A

In Problem 4, we put K=L=3, $G=B_{K,L}\cup \left\{\left(1,1\right),\left(2,2\right)\right\}$ (see Figure A1).

The task can be formulated as follows:

$$\begin{array}{cc} \max_{n_{i,j}}\;\sum_{i=1}^{3}\sum_{j=1}^{3}-n_{i,j}\ln & \frac{n_{i,j}}{a_{i}b_{j}}\;,\;i,j\in \left\{1;2;3\right\}\;,\\ \mathrm{under}\mathrm{conditions}\;n_{i,j} & \geq 0\;,\\ n_{1,1} & =z_{1,1}\;,\\ n_{1,1}+n_{2,1}+n_{3,1} & =z_{3,1}\;,\\ n_{1,1}+n_{2,1}+n_{1,2}+n_{2,2} & =z_{2,2}\;,\\ n_{1,1}+n_{2,1}+n_{3,1}+n_{1,2}+n_{2,2}+n_{3,2} & =z_{3,2}\;,\\ n_{1,1}+n_{1,2}+n_{1,3} & =z_{1,3}\;,\\ n_{1,1}+n_{2,1}+n_{1,2}+n_{2,2}+n_{1,3}+n_{2,3} & =z_{2,3}\;,\\ n_{1,1}+n_{2,1}+n_{3,1}+n_{1,2}+n_{2,2}+n_{3,2}+n_{1,3}+n_{2,3}+n_{3,3} & =z_{3,3}\;. \end{array}$$

We take $n_{2,2}$ and $n_{2,3}$ as variables, allowing us to express the general solution of the system of linear equations; we introduce new constants $C_{*}$ as abbreviations:

$$\begin{array}{cc} n_{1,1} & =z_{1,1}=C_{1}\;,\\ n_{2,1} & =-n_{2,2}-n_{2,3}\underset{C_{2}}{\underbrace{-z_{1,3}+z_{2,3}}}=-n_{2,2}-n_{2,3}+C_{2}\;,\\ n_{3,1} & =n_{2,2}+n_{2,3}\underset{C_{3}}{\underbrace{-z_{1,1}+z_{3,1}+z_{1,3}-z_{2,3}}}=n_{2,2}+n_{2,3}+C_{3}\;,\;\;\;\;\;\;\;\;\;\;\;\;\;\;\;\;\;\;\;\;\;\;\;\;\;\;\;\\ n_{1,2} & =n_{2,3}\underset{C_{4}}{\underbrace{-z_{1,1}+z_{2,2}+z_{1,3}-z_{2,3}}}=n_{2,3}+C_{4}\;,\\ n_{3,2} & =-n_{2,2}-n_{2,3}\underset{C_{5}}{\underbrace{+z_{1,1}-z_{3,1}-z_{2,2}+z_{3,2}-z_{1,3}+z_{2,3}}}=-n_{2,2}-n_{2,3}+C_{5}\;,\\ n_{1,3} & =-n_{2,3}\underset{C_{6}}{\underbrace{-z_{2,2}+z_{2,3}}}=-n_{2,3}+C_{6}\;,\\ n_{3,3} & =\underset{C_{7}}{\underbrace{z_{2,2}-z_{3,2}-z_{2,3}+z_{3,3}}}=C_{7}\;. \end{array}$$

Substituting this in the optimality criterion and putting its partial derivatives equal to zero, we obtain

$$\begin{array}{cc} \; & \ln\frac{C_{2}-n_{2,2}-n_{2,3}}{a_{2}b_{1}}+1-\ln\frac{C_{3}+n_{2,2}+n_{2,3}}{a_{3}b_{1}}-1-\ln\frac{n_{2,2}}{a_{2}b_{2}}-1\\ + & \ln\frac{C_{5}-n_{2,2}-n_{2,3}}{a_{3}b_{2}}+1=0\;,\\ \; & \ln\frac{C_{2}-n_{2,2}-n_{2,3}}{a_{2}b_{1}}+1-\ln\frac{C_{3}+n_{2,2}+n_{2,3}}{a_{3}b_{1}}-1-\ln\frac{C_{4}+n_{2,3}}{a_{1}b_{2}}-1\\ + & \ln\frac{C_{5}-n_{2,2}-n_{2,3}}{a_{3}b_{2}}+1+\ln\frac{C_{6}-n_{2,3}}{a_{1}b_{3}}+1-\ln\frac{n_{2,3}}{a_{2}b_{3}}-1=0\;. \end{array}$$

These equations can be simplified to

$$\begin{array}{cc} \ln\frac{\left(C_{2}-n_{2,2}-n_{2,3}\right)\left(C_{5}-n_{2,2}-n_{2,3}\right)}{a_{2}b_{1}a_{3}b_{2}} & =\ln\frac{\left(C_{3}+n_{2,2}+n_{2,3}\right)n_{2,2}}{a_{3}b_{1}a_{2}b_{2}}\;,\\ \ln\frac{\left(C_{2}-n_{2,2}-n_{2,3}\right)\left(C_{5}-n_{2,2}-n_{2,3}\right)\left(C_{6}-n_{2,3}\right)}{a_{2}b_{1}a_{3}b_{2}a_{1}b_{3}} & =\ln\frac{\left(C_{3}+n_{2,2}+n_{2,3}\right)\left(C_{4}+n_{2,3}\right)n_{2,3}}{a_{3}b_{1}a_{1}b_{2}a_{2}b_{3}}\;. \end{array}$$

The divisors are equal and thus cancel out, as shown in the proof of Theorem 1, and this leads to the expression

(A1) $$\begin{array}{cc} \left(C_{2}-n_{2,2}-n_{2,3}\right)\left(C_{5}-n_{2,2}-n_{2,3}\right) & =\left(C_{3}+n_{2,2}+n_{2,3}\right)n_{2,2}\;,\\ \left(C_{2}-n_{2,2}-n_{2,3}\right)\left(C_{5}-n_{2,2}-n_{2,3}\right)\left(C_{6}-n_{2,3}\right) & =\left(C_{3}+n_{2,2}+n_{2,3}\right)\left(C_{4}+n_{2,3}\right)n_{2,3}\;. \end{array}$$

We divide the second equation by the first

$$C_{6}-n_{2,3}=\frac{\left(C_{4}+n_{2,3}\right)n_{2,3}}{n_{2,2}}\;,$$

and $n_{2,2}$ can be expressed as

(A2) $$n_{2,2}=\frac{\left(C_{4}+n_{2,3}\right)n_{2,3}}{\left(C_{6}-n_{2,3}\right)}\;.$$

Substituting into (A1) and expanding, we obtain an equation for $n_{2,3}$,

$$\begin{array}{c} \left(C_{3}-C_{4}-C_{6}\right)n_{2,3}^{3}\\ +\left(C_{2}C_{6}+C_{2}C_{4}+C_{2}C_{5}+C_{6}^{2}+C_{4}C_{6}+C_{5}C_{6}-C_{3}C_{6}+C_{4}C_{5}+C_{3}C_{4}\right)n_{2,3}^{2}\\ +\left(-C_{2}C_{6}^{2}-C_{2}C_{4}C_{6}-C_{2}C_{5}C_{6}-C_{5}C_{6}^{2}-C_{4}C_{5}C_{6}-C_{3}C_{4}C_{6}\right)n_{2,3}\\ +C_{2}C_{5}C_{6}^{2}=0\;. \end{array}$$

This is an algebraic equation of order three. It can be solved analytically using Cardano formulas. A problem remains that it can have up to three roots; our objective is to obtain one that is real. The cubic coefficient is

$$C_{3}-C_{4}-C_{6}=z_{3,1}-z_{2,3}\;.$$

If it is nonzero, there is at least one real root. Moreover, we need it in the interval [0,1]. Substituting to (A2), we obtain $n_{2,2}$, which should also be in the interval [0,1], as well as all other unknowns. However, we know (from the convexity of the task, resp., concavity of the maximized criterion) that a solution with this property exists.

## Appendix B

In Problem 4, we put K=3, L=4, $G=B_{K,L}\cup \left\{\left(1,2\right),\left(2,1\right),\left(2,3\right)\right\}$ (see Figure A2).

The task can be formulated as follows:

$$\begin{array}{cc} \max_{n_{i,j}}\;\sum_{i=1}^{3}\sum_{j=1}^{4}-n_{i,j}\ln & \frac{n_{i,j}}{a_{i}b_{j}}\;,\;i\in \left\{1;2;3\right\}\;,\;j\in \left\{1;2;3;4\right\}\;,\\ \mathrm{under}\mathrm{conditions}\;n_{i,j} & \geq 0\;,\\ n_{2,1} & =C_{1}-n_{1,1}\;,\\ n_{1,2} & =C_{2}-n_{1,1}\;,\\ n_{3,2} & =C_{3}+n_{1,1}-n_{2,2}\;,\\ n_{2,3} & =C_{4}+n_{1,1}-n_{2,2}-n_{1,3}\;,\\ n_{3,3} & =C_{5}-n_{1,1}+n_{2,2}\;,\\ n_{1,4} & =C_{6}\;\;\;\;\;\;-n_{1,3}\;,\\ n_{2,4} & =C_{7}\;\;\;\;\;\;+n_{1,3}\;, \end{array}$$

where the constants $C_{*}$ are some sums of $\pm z_{i,j}$ as in the previous example. We take $n_{1,1}$, $n_{2,2}$, and $n_{1,3}$ as variables, allowing us to express the general solution of the system of linear equations.

We put the partial derivatives equal to zero and eliminate the logarithms. We obtain products of factors in the form of a sum of some $\pm n_{i,j}$ and a constant $C_{*}$. We also know that the result is independent of the values $a_{i}$, $b_{j}$. In particular, we obtain

(A3) $$\begin{array}{cc} \frac{\partial }{\partial n_{1,1}}:\; & \left(C_{1}-n_{1,1}\right)\left(C_{2}-n_{1,1}\right)\left(C_{5}-n_{1,1}+n_{2,2}\right)\\ \; & =n_{1,1}\left(C_{3}+n_{1,1}-n_{2,2}\right)\left(C_{4}+n_{1,1}-n_{2,2}-n_{1,3}\right)\;,\\ \frac{\partial }{\partial n_{2,2}}:\; & \left(C_{3}+n_{1,1}-n_{2,2}\right)\left(C_{4}+n_{1,1}-n_{2,2}-n_{1,3}\right)=\left(C_{5}-n_{1,1}+n_{2,2}\right)n_{2,2}\;,\\ \frac{\partial }{\partial n_{1,3}}:\; & \left(C_{4}+n_{1,1}-n_{2,2}-n_{1,3}\right)\left(C_{6}-n_{1,3}\right)=n_{1,3}\left(C_{7}+n_{1,3}\right)\;. \end{array}$$

From (A3), we separate the factors containing only the variable $n_{1,1}$

(A4) $$\frac{\left(C_{1}-n_{1,1}\right)\left(C_{2}-n_{1,1}\right)}{n_{1,1}}=\frac{\left(C_{3}-n_{2,2}+n_{1,1}\right)\left(C_{4}+n_{1,1}-n_{2,2}-n_{1,3}\right)}{C_{5}-n_{1,1}+n_{2,2}}\;.$$

The second equation in (A3) can be written as

(A5) $$\frac{\left(C_{3}-n_{2,2}+n_{1,1}\right)\left(C_{4}+n_{1,1}-n_{2,2}-n_{1,3}\right)}{C_{5}-n_{1,1}+n_{2,2}}=n_{2,2}\;.$$

We substitute (A4) to the left-hand side and express $n_{2,2}$ as

(A6) $$n_{2,2}=\frac{\left(C_{1}-n_{1,1}\right)\left(C_{2}-n_{1,1}\right)}{n_{1,1}}\;.$$

The third equation in (A3) can be rewritten as

(A7) $$\begin{array}{cc} C_{4}+n_{1,1}-n_{2,2}-n_{1,3} & =\frac{n_{1,3}\left(C_{7}+n_{1,3}\right)}{C_{6}-n_{1,3}}\;,\\ C_{4}+n_{1,1}-n_{2,2} & =\frac{n_{1,3}\left(C_{7}+n_{1,3}\right)+n_{1,3}\left(C_{6}-n_{1,3}\right)}{C_{6}-n_{1,3}}\;,\\ C_{4}+n_{1,1}-n_{2,2} & =\frac{n_{1,3}\left(C_{7}+C_{6}\right)}{C_{6}-n_{1,3}}\;,\\ C_{4}+n_{1,1}-n_{2,2} & =-\left(C_{7}+C_{6}\right)+\frac{\left(C_{7}+C_{6}\right)C_{6}}{C_{6}-n_{1,3}}\;. \end{array}$$

In the last equation of (A7), we substitute $n_{2,2}$ from (A6) and obtain $n_{1,3}$:

(A8) $$\begin{array}{cc} C_{4}+n_{1,1}-\frac{\left(C_{1}-n_{1,1}\right)\left(C_{2}-n_{1,1}\right)}{n_{1,1}}=-\left(C_{7}+C_{6}\right)+ & \frac{\left(C_{7}+C_{6}\right)C_{6}}{C_{6}-n_{1,3}}\;,\\ \frac{\left(C_{4}+n_{1,1}\right)n_{1,1}-\left(C_{1}-n_{1,1}\right)\left(C_{2}-n_{1,1}\right)+\left(C_{7}+C_{6}\right)n_{1,1}}{n_{1,1}} & =\frac{\left(C_{7}+C_{6}\right)C_{6}}{C_{6}-n_{1,3}}\;,\\ \frac{n_{1,1}\left(C_{1}+C_{2}+C_{4}+C_{6}+C_{7}\right)-C_{1}C_{2}}{n_{1,1}} & =\frac{\left(C_{7}+C_{6}\right)C_{6}}{C_{6}-n_{1,3}}\;,\\ \frac{n_{1,1}}{n_{1,1}\left(C_{1}+C_{2}+C_{4}+C_{6}+C_{7}\right)-C_{1}C_{2}} & =\frac{C_{6}-n_{1,3}}{\left(C_{7}+C_{6}\right)C_{6}}\;,\\ \frac{n_{1,1}\left(C_{7}+C_{6}\right)C_{6}}{n_{1,1}\left(C_{1}+C_{2}+C_{4}+C_{6}+C_{7}\right)-C_{1}C_{2}} & =C_{6}-n_{1,3}\;,\\ \frac{C_{6}\left(n_{1,1}\left(C_{1}+C_{2}+C_{4}+C_{6}+C_{7}\right)-C_{1}C_{2}\right)-n_{1,1}\left(C_{7}+C_{6}\right)C_{6}}{n_{1,1}\left(C_{1}+C_{2}+C_{4}+C_{6}+C_{7}\right)-C_{1}C_{2}} & =n_{1,3}\;,\\ \frac{C_{6}\left(n_{1,1}\left(C_{1}+C_{2}+C_{4}\right)-C_{1}C_{2}\right)}{n_{1,1}\left(C_{1}+C_{2}+C_{4}+C_{6}+C_{7}\right)-C_{1}C_{2}} & =n_{1,3}\;. \end{array}$$

We have both $n_{2,2}$ in (A6) and $n_{1,3}$ in (A8) expressed as functions of $n_{1,1}$. This can be substituted to the first equation in (A3), which yields

(A9) $$\begin{array}{cc} L:\; & \left(C_{1}-n_{1,1}\right)\left(C_{2}-n_{1,1}\right)\left(C_{5}-n_{1,1}+\frac{\left(C_{1}-n_{1,1}\right)\left(C_{2}-n_{1,1}\right)}{n_{1,1}}\right)\;,\\ R:\; & n_{1,1}\left(C_{3}-\frac{\left(C_{1}-n_{1,1}\right)\left(C_{2}-n_{1,1}\right)}{n_{1,1}}+n_{1,1}\right)(C_{4}+n_{1,1}-\frac{\left(C_{1}-n_{1,1}\right)\left(C_{2}-n_{1,1}\right)}{n_{1,1}}\\ \; & -\frac{C_{6}\left(n_{1,1}\left(C_{1}+C_{2}+C_{4}\right)-C_{1}C_{2}\right)}{n_{1,1}\left(C_{1}+C_{2}+C_{4}+C_{6}+C_{7}\right)-C_{1}C_{2}})\;, \end{array}$$

where *L* (resp., *R*) denotes the left-hand (resp., right-hand) side of the equation. We modify this expression to discuss its analytical solvability:

(A10) $$\begin{array}{cc} \; & L:\;\frac{\left(n_{1,1}^{2}-\left(C_{1}+C_{2}\right)n_{1,1}+C_{1}C_{2}\right)\left(\left(-C_{1}-C_{2}+C_{5}\right)n_{1,1}-C_{1}C_{2}\right)}{n_{1,1}}\;,\\ \; & R:\;(\left(C_{1}+C_{2}+C_{3}\right)n_{1,1}-C_{1}C_{2})\\ \; & \cdot \frac{\left(n_{1,1}^{2}\left(C_{1}+C_{2}+C_{4}\right)\left(C_{1}+C_{2}+C_{4}+C_{7}\right)-n_{1,1}C_{1}C_{2}\left(2C_{1}+2C_{2}+2C_{4}+2C_{6}+C_{7}\right)+C_{1}^{2}C_{2}^{2}\right)}{n_{1,1}\left(n_{1,1}\left(C_{1}+C_{2}+C_{4}+C_{6}+C_{7}\right)-C_{1}C_{2}\right)}\;. \end{array}$$

Multiplication by a common divisor of both sides gives

(A11) $$\begin{array}{cc} L:\; & \left(n_{1,1}^{2}-\left(C_{1}+C_{2}\right)n_{1,1}+C_{1}C_{2}\right)\left(\left(-C_{1}-C_{2}+C_{5}\right)n_{1,1}-C_{1}C_{2}\right)\\ \; & \cdot (\left(C_{1}+C_{2}+C_{4}+C_{6}+C_{7}\right)n_{1,1}-C_{1}C_{2})\;,\\ R:\; & \left(\left(C_{1}+C_{2}+C_{3}\right)n_{1,1}-C_{1}C_{2}\right)(n_{1,1}^{2}\left(C_{1}+C_{2}+C_{4}\right)\left(C_{1}+C_{2}+C_{4}+C_{7}\right)\\ \; & -n_{1,1}C_{1}C_{2}\left(2C_{1}+2C_{2}+2C_{4}+2C_{6}+C_{7}\right)+C_{1}^{2}C_{2}^{2})\;. \end{array}$$

After expansion, the left-hand side is a polynomial of order 4 and the right-hand side is a polynomial of order 3. Their difference is a polynomial of order 4, which is solvable analytically.

The remaining variables are obtained by substitution to (A6) and (A8). This results in the set of all solutions; substitution of the feasible solution to the equation for differential entropy allows us to determine the maximum.
